# Supplementary material for: Attitudes towards free-roaming dogs and dog ownership practices in Bulgaria, Italy, and Ukraine
Source: PLoS One. 2022 Mar 2;17(3):e0252368. doi: 10.1371/journal.pone.0252368 (PMC8890656; doi:10.1371/journal.pone.0252368)
Supplement: S1 File — (DOCX) [file pone.0252368.s001.docx]

**Supporting information**

1. Facebook adverts:

Four Facebook adverts were used to increase recruitment of participants to complete the questionnaire. The adverts in English were:

Advert 1:

Headline 1: We would like to hear your opinion on stray dogs!

You are being invited to participate in this research project as we are looking to recruit a wide range of people from many different backgrounds so that we can have a clear idea of public attitudes towards stray dogs.

Advert 2:

Headline 2: Your opinion on stray dogs- take the survey!

We are conducting a study on the stray dog populations in Europe and internationally. Stray dog overpopulation is a global problem which is of public health, animal welfare and environmental concern. Let us know your opinion on stray dogs.

Advert 3:

Headline 3: Volunteers Required

We would like to hear your opinion about your local stray dog problem! Help us now by filling out our questionnaire.

Advert 4:

Headline 4: Assist us by letting us know your opinion

The STRAYS project investigates and compares different methods for long-term stray dog population management through a series of computer simulations. This will allow us to directly quantify the long-term effectiveness and sustainability of the catch-neuter-release method compared to other stray dog population management methods (including sheltering and culling).
