# Supplementary material for: Attitudes towards free-roaming dogs and dog ownership practices in Bulgaria, Italy, and Ukraine
Source: PLoS One. 2022 Mar 2;17(3):e0252368. doi: 10.1371/journal.pone.0252368 (PMC8890656; doi:10.1371/journal.pone.0252368)
Supplement: S2 File — (DOCX) [file pone.0252368.s002.docx]

**S2 File**

1. English copy of questionnaire

You are being invited to participate in a research study titled “Investigating the sustainability of the Catch-Neuter-Release programme for the control of stray dog populations”. This study is being carried out by a team at the University of Leeds in the United Kingdom, VIER PFOTEN International and Istituto Zooprofilattico Sperimentale Dell’Abruzzo e del Molise (IZSAM), Italy. The purpose of this research is to gather information about the attitudes towards stray dogs globally. This study will take between 5 to 10 minutes to complete. Your participation is entirely voluntary and you are free to withdraw from the questionnaire at any point and do not have to give a reason. You do not have to answer any questions you do not want to.

Your participation in this study will remain confidential, and only anonymised data will be published. We will minimise any risks by ensuring that your answers will remain completely anonymous.

1) I confirm that: (i) I have read and understood the information sheet explaining the research project; (ii) I understand I have the opportunity to ask questions about the project; and (iii) i know that I can withdraw from completing the questionnaire, prior to submitting. 2) I understand that as this data is collected anonymously I will not be able to withdraw from this study after my responses have been submitted. 3) I agree for the data collected from me to be stored and used in relevant future research in an anonymised form. 4) I agree that the University of Leeds and the project partners are not liable for any negative consequences, unless found to be negligent. *Required

Yes/No

**Section 1) Socio-demographic information**

1. What region do you live in?
2. What is your age?

| Under 18 |  |
| --- | --- |
| 18 to 24 |  |
| 25 to 34 |  |
| 35 to 44 |  |
| 45 to 54 |  |
| 55 to 64 |  |
| 65 to 74 |  |
| 75 and over |  |
| No answer |  |

1. Which gender do you identify as?

| Male |  |
| --- | --- |
| Female |  |
| Non-binary |  |
| Other (please state) |  |
| No answer |  |

Other: ……………………………………………………………

1. What is your occupation?

| Employed |  |
| --- | --- |
| Unemployed |  |
| Student |  |
| Seeking work |  |
| Housewife/husband |  |
| Retired |  |
| No answer |  |
| Other (please state) |  |

Other: ……………………………………………………………

1. Education status:

| No schooling |  |
| --- | --- |
| Primary education (between ages 5 and 12) |  |
| Secondary education (between ages 11 and 18) |  |
| Higher education (University or colleges) |  |
| No answer |  |

1. Nationality:

…………………………………………..

1. Religious beliefs

Atheist, Agnostic, Baha'i, Buddhist, Candomblé, Christian, Hindu, Jain, Jehovah's Witnesses, Jewish, Mormon, Muslim, Paganism, Rastafarian, Santeria, Shinto, Sikh, Spiritualist, Taoism, Unitarianism, Zoroastrianism, No answer,

Other: ……………………………………………………………

1. Relationship status

| Single |  |
| --- | --- |
| Married |  |
| Cohabitating |  |
| Divorced/widowed |  |
| No answer |  |

1. Number of people living in household:

| 1 | 2 | 3 | 4 | 5 | More than 5 | No answer |
| --- | --- | --- | --- | --- | --- | --- |
|  |  |  |  |  |  |  |

1. Number of children in household

| 1 | 2 | 3 | 4 | 5 | More than 5 | No answer |
| --- | --- | --- | --- | --- | --- | --- |
|  |  |  |  |  |  |  |

**Section 2) Dog Ownership Practices**

1. Do you own a dog?

| Yes |  |
| --- | --- |
| No |  |
| No answer |  |

If no, please skip to **section 3**. If yes, continue in **section 2**.

1. How many male dogs do you own?

| 0 | 1 | 2 | 3 | 4 | 5 | More than 5 | No answer |
| --- | --- | --- | --- | --- | --- | --- | --- |
|  |  |  |  |  |  |  |  |

1. How many female dogs do you own?

| 0 | 1 | 2 | 3 | 4 | 5 | More than 5 | No answer |
| --- | --- | --- | --- | --- | --- | --- | --- |
|  |  |  |  |  |  |  |  |

1. How many of your dog(s) are:

| Under 1 year old | 1 to 3 years old | Over 3 years old | No answer |
| --- | --- | --- | --- |
|  |  |  |  |

1. Are your dogs registered and identified?

| Yes |  |
| --- | --- |
| No |  |
| I don’t know |  |
| No answer |  |

1. What is your main reason for owning a dog(s)? Please select as many as you wish.

| For practical reasons e.g. to guard house or for hunting |  |
| --- | --- |
| For pleasure and company e.g. as a pet or companion |  |
| No answer |  |
| Other (please state) |  |

Other: ……………………………………………………………

1. Where did you get your dog(s)?

| A dog shelter |  |
| --- | --- |
| Internet |  |
| A shop |  |
| A breeder |  |
| Bred my own |  |
| Found |  |
| From a friend/family |  |
| No answer |  |
| Other (please state) |  |

Other: ……………………………………………………………

1. Did you pay for your dog?

| Yes |  |
| --- | --- |
| No |  |
| No answer |  |

1. How old was/were your dog(s) when you got him/her?

| Puppy |  |
| --- | --- |
| Adult |  |
| No answer |  |

1. Have any of your male or female dog(s) had puppies?

| Yes |  |
| --- | --- |
| No |  |
| Don’t know |  |
| No answer |  |

If **yes**,

1. Considering all of your dogs, in total how many times have your dog(s) had puppies?

| Once |  |
| --- | --- |
| Twice |  |
| Three times or more |  |
| No answer |  |

1. What did you do with the puppies? Please mark all that apply.

| Kept the puppies |  |
| --- | --- |
| Gave them to a shelter |  |
| Phoned authorities |  |
| Gave to a friend |  |
| Sold the puppies |  |
| Let them free in the street |  |
| Euthanised them at a clinic |  |
| No answer |  |
| Other (please state) |  |

Other: ……………………………………………………………

1. Do you prevent your dog(s) from breeding?

| Yes |  |
| --- | --- |
| No |  |
| No answer |  |
| Other |  |

Other: ……………………………………………………………

1. If **yes**, how do you prevent your dog(s) from breeding?

| Surgical neutering |  |
| --- | --- |
| Restricting male and female contact |  |
| No answer |  |
| Other (please state) |  |

Other: ……………………………………………………………

1. If **no**, what is your main reason for not preventing your dog(s) from breeding?

| Cost |  |
| --- | --- |
| A dog should reproduce at least once |  |
| Believe dog is too young to be neutered |  |
| Neutering is against religious beliefs |  |
| Neutering causes weight gain |  |
| Neutering modifies the dog’s behaviour |  |
| Neutering is a risk to the dog’s health |  |
| No answer |  |
| Other (please state) |  |

Other: ……………………………………………………………

1. Do you feed your dog every day?

| Yes |  |
| --- | --- |
| No |  |
| No answer |  |

1. Do you give your dog water every day?

| Yes |  |
| --- | --- |
| No |  |
| No answer |  |

1. Do you provide shelter for your dog every day?

| Yes |  |
| --- | --- |
| No |  |
| No answer |  |

1. Do you vaccinate your dog(s)?

| Yes |  |
| --- | --- |
| No |  |
| No answer |  |

1. Do you allow your dog(s) to go outside, in the street, unsupervised?

| Always |  |
| --- | --- |
| Sometimes |  |
| Never |  |
| No answer |  |

1. Have you ever given up a dog?

| Yes |  |
| --- | --- |
| No |  |
| No answer |  |

If no, go to question 33

1. If yes, did you:

| Give the dog to a shelter |  |
| --- | --- |
| Phone authorities |  |
| Give to friend |  |
| Sell |  |
| Let free |  |
| Euthanise at a clinic |  |
| No answer |  |
| Other (please state) |  |

Other: ……………………………………………………………

1. If yes, what was your reason for giving up a dog?

| Lost interest |  |
| --- | --- |
| Animal behavioural problem |  |
| Cost |  |
| No answer |  |
| Other (please state) |  |

Other: ……………………………………………………………

**Section 3) Attitudes Towards Stray Dogs**

1. Have you seen dogs free on the street

| Today |  |
| --- | --- |
| In the past week |  |
| In the past month |  |
| In the past year |  |
| Never |  |
| No answer |  |

1. Have you ever felt physically threatened by dogs in the street?

| Yes |  |
| --- | --- |
| No |  |
| No answer |  |

1. Have you ever been attacked by dogs in the street?

| Yes |  |
| --- | --- |
| No |  |
| No answer |  |

1. Have you or any of your family members been bitten by dogs in the street in the last 12 months?

| Yes |  |
| --- | --- |
| No |  |
| No answer |  |

1. Do you ever provide care for free-roaming dogs by (please choose as many as necessary):

| Feeding |  |
| --- | --- |
| Providing water |  |
| Providing shelter |  |
| None |  |
| No answer |  |

Please select your level of agreement with the following statements:

1. **I do not like free-roaming dogs being present in the streets around my home or work.**

| Strongly agree | Agree | Neither agree nor disagree | Disagree | Strongly disagree | No answer |
| --- | --- | --- | --- | --- | --- |
|  |  |  |  |  |  |

1. **It is a good thing for the public to provide shelter for free-roaming dogs.**

| Strongly agree | Agree | Neither agree nor disagree | Disagree | Strongly disagree | No answer |
| --- | --- | --- | --- | --- | --- |
|  |  |  |  |  |  |

1. **It is unacceptable for the public to feed free-roaming dogs*.***

| Strongly agree | Agree | Neither agree nor disagree | Disagree | Strongly disagree | No answer |
| --- | --- | --- | --- | --- | --- |
|  |  |  |  |  |  |

1. **I feel physically threatened by free-roaming dogs.**

| Strongly agree | Agree | Neither agree nor disagree | Disagree | Strongly disagree | No answer |
| --- | --- | --- | --- | --- | --- |
|  |  |  |  |  |  |

1. **Stray dogs spread diseases.**

| Strongly agree | Agree | Neither agree nor disagree | Disagree | Strongly disagree | No answer |
| --- | --- | --- | --- | --- | --- |
|  |  |  |  |  |  |

1. **Stray dogs are a threat to the safety of children.**

| Strongly agree | Agree | Neither agree nor disagree | Disagree | Strongly disagree | No answer |
| --- | --- | --- | --- | --- | --- |
|  |  |  |  |  |  |

1. **Stray dogs spread rubbish and faeces**

| Strongly agree | Agree | Neither agree nor disagree | Disagree | Strongly disagree | No answer |
| --- | --- | --- | --- | --- | --- |
|  |  |  |  |  |  |

1. **It is unacceptable for the public to provide water for free-roaming dogs.**

| Strongly agree | Agree | Neither agree nor disagree | Disagree | Strongly disagree | No answer |
| --- | --- | --- | --- | --- | --- |
|  |  |  |  |  |  |

1. Who do you think should be responsible for managing free-roaming dogs (such as by providing care and/or preventing an increase in free-roaming dogs)? Pick the top three.

| National government |  |
| --- | --- |
| Municipality government |  |
| Public veterinarians |  |
| Private veterinarians |  |
| Police |  |
| Volunteer organisations |  |
| Garbage control |  |
| Nobody |  |
| No answer |  |
| Other (please state) |  |

Other: ……………………………………………………………

1. Do you think an increase in free-roaming dogs should be prevented?

| Yes |  |
| --- | --- |
| No |  |
| No answer |  |

1. If **yes**, how do you think free-roaming dogs should be prevented (please pick as many as necessary)?

| Public education campaigns for responsible dog ownership |  |
| --- | --- |
| School education campaigns |  |
| Sanctions for abandoning dogs |  |
| No answer |  |
| Other (please state) |  |

Other: ……………………………………………………………

1. Would you prefer to see:

| No free-roaming dogs | Fewer free-roaming dogs | You do not mind free-roaming dogs | More free-roaming dogs | No answer |
| --- | --- | --- | --- | --- |
|  |  |  |  |  |

1. If you would prefer to see no dogs or fewer dogs on the street, how do you think free-roaming dogs should be reduced (please pick as many as necessary)?

| Remove dogs and put in shelters |  |
| --- | --- |
| Catch-neuter-return of free-roaming dogs |  |
| Controlling the birth rate of owned dogs |  |
| Culling |  |
| No answer |  |
| I do not mind dogs on the street |  |
| Other (please state) |  |

Other: ……………………………………………………………

Information Sheet

**Research Project Title**

Investigating the sustainability of the Catch-Neuter-Release programme for the control of stray dog populations.

**Research Project Description**

This research project aims to determine whether neutering stray dogs (e.g. sterilisation through spay or castration) is a sustainable method of lessening the number of stray dogs on the street, when compared to other methods such as culling or moving dogs into shelter.

**Invitation to participate**

You are being invited to participate in this research project as we are looking to recruit a wide range of people from many different backgrounds so that we can have a clear idea of public attitudes towards stray dogs. The reasons why this research is being carried out and what will be involved are described below. Please take time to read the following information carefully. If any part is unclear or if you would like more information, please ask us (contact details below).

**Purpose of the project**

This study is part of a multinational stray dog population control project carried out by the University of Leeds in the United Kingdom and scientific collaborators VIER PFOTEN International and Istituto Zooprofilattico Sperimentale Dell’Abruzzo e del Molise. There are a large number of stray dogs globally. This project would like to find out the most sustainable way to decrease the number of stray dogs while taking into consideration cost, public health risk and the welfare implications of the methods used. This is a three-year study as part of a PhD project at the University of Leeds.

**Do I have to take part?**

Taking part in this study is entirely voluntary. You may discontinue participation at any point of the questionnaire and you do not have to give a reason. If you decide to take part, you will be asked for your consent. However, please be aware that as this data is collected anonymously, once responses have been submitted your participation in this study cannot be withdrawn. You may save this information sheet to refer to at a later date.

**What do I have to do? / What will happen to me if I take part?**

You will be asked to complete a questionnaire which will take approximately 5 to 10 minutes.

**Possible disadvantages and risks of taking part?**

There are no foreseeable risks in participating in this questionnaire. All responses will remain anonymous*.*

**What are the possible benefits of taking part?**

Whilst there are no immediate benefits for those people participating in the project, it is hoped that this work will help inform stray dog population control programmes.

**What type of information will be sought from me and why is the collection of this information relevant for achieving the research project’s objectives?**

Information regarding your dog ownership practices and attitudes towards the stray dogs will be collected. This will allow us to find out more about public attitudes towards stray dogs and help inform stray dog management.

**Confidentiality and anonymity**

All the information that we collect about you during the course of the research will be kept strictly anonymous. We will not collect any directly identifiable information, such as your name.

This information will be written up in as part of a PhD thesis, shared in reports to project collaborators and in publications to the wider scientific community. You will not be identified in any of the reports or as a result of publishing the data. If you would like to know the results of the study, please contact [STRAYS@leeds.ac.uk](mailto:STRAYS@leeds.ac.uk).

**Who is organising/funding the research?**

This research is funded by VIER PFOTEN International and collaborates with Istituto Zooprofilattico Sperimentale dell’Abruzzo e del Molise (IZSAM) and the University of Leeds, UK.

**What if there is a problem?**

If you are concerned about any aspect of the questionnaire, please contact the researchers (contact details below), who will do their best to answer your question. In the unlikely event that something does go wrong, the University of Leeds and the collaborators (VIER PFOTEN International and IZSAM) are not liable for any negative consequences, unless found to be negligent.

**Contact for further information**

For further information, please contact: [STRAYS@leeds.ac.uk](mailto:bslsmi@leeds.ac.uk).

Thank you very much for taking the time to read through this information.
