# Supplementary material for: Attitudes towards free-roaming dogs and dog ownership practices in Bulgaria, Italy, and Ukraine
Source: PLoS One. 2022 Mar 2;17(3):e0252368. doi: 10.1371/journal.pone.0252368 (PMC8890656; doi:10.1371/journal.pone.0252368)
Supplement: S3 File — (DOCX) [file pone.0252368.s003.docx]

**S3 File**

Answer option to question “Are your dog(s) registered *and identified*” in Bulgarian questionnaire:

For the question “*Are your dog(s) registered and identified*” in the Bulgarian questionnaire the answer option “*No – none of them*” was not included due to a translation error. As this answer is not included in any statistical analysis, I present the descriptive results for the other answer options, which are the same as the options available for the Italian and Ukrainian questionnaires.
