# Supplementary material for: Attitudes towards free-roaming dogs and dog ownership practices in Bulgaria, Italy, and Ukraine
Source: PLoS One. 2022 Mar 2;17(3):e0252368. doi: 10.1371/journal.pone.0252368 (PMC8890656; doi:10.1371/journal.pone.0252368)
Supplement: S1 Table — (DOCX) [file pone.0252368.s004.docx]

S1 Table

Number of no responses

S1 Table. Number of "No responses" to outcome and predictor variables in statistical analysis.

| **Dataset** | **Variable** | **No. NA's** | **Total number of respondents** | **%** |
| --- | --- | --- | --- | --- |
| Dog owners | *Neutering status of owned dogs* | 0 | 16906 | 0.0% |
|  | *Roaming status of owned dogs* | 168 |  | 1.0% |
|  | *Gender* | 71 |  | 0.4% |
|  | *Age* | 120 |  | 0.7% |
|  | *Education status* | 843 |  | 5.0% |
|  | *Religious belief* | 2256 |  | 13.3% |
|  | *Owning a dog for practical reasons* | 0 |  | 0.0% |
|  | *Country* | 0 |  | 0.0% |
| All | *I do not like the presence of free-roaming dogs around my home or work* | 2870 | 28225 | 10.2% |
|  | *Should an increase in dogs on the street be prevented* | 662 |  | 2.3% |
|  | *Would you prefer to see dogs on the street?* | 329 |  | 1.2% |
|  | *Child in household* | 549 |  | 1.9% |
|  | *Feel physically threatened by dogs on the street* | 2320 |  | 8.2% |
|  | *Been attacked by dogs on the street* | 303 |  | 1.1% |
|  | *Respondent of family members have been bitten by dogs on the street in the last 12 months* | 364 |  | 1.3% |
|  | *Country* | 0 |  | 0.0% |
