# Supplementary material for: Attitudes towards free-roaming dogs and dog ownership practices in Bulgaria, Italy, and Ukraine
Source: PLoS One. 2022 Mar 2;17(3):e0252368. doi: 10.1371/journal.pone.0252368 (PMC8890656; doi:10.1371/journal.pone.0252368)
Supplement: S2 Table — (DOCX) [file pone.0252368.s005.docx]

Descriptive results of questionnaire

S2 Table. Demographic information about respondents in Bulgaria, Italy and Ukraine.

|  | **Bulgaria** | **%** | **Italy** | **%** | **Ukraine** | **%** |
| --- | --- | --- | --- | --- | --- | --- |
| **Total respondents** | **5434** |  | **3468** |  | **19323** |  |
| **Age** |  |  |  |  |  |  |
| 18 - 24 | 1172 | 21.6 | 852 | 24.6 | 3742 | 19.4 |
| 25 - 34 | 1209 | 22.2 | 648 | 18.7 | 6049 | 31.3 |
| 35 - 44 | 1011 | 18.6 | 378 | 10.9 | 3983 | 20.6 |
| 45 - 54 | 1112 | 20.5 | 573 | 16.5 | 3269 | 16.9 |
| 55 - 64 | 658 | 12.1 | 729 | 21.0 | 1764 | 9.1 |
| 65 - 74 | 211 | 3.9 | 229 | 6.6 | 368 | 1.9 |
| 75 and over | 11 | 0.2 | 30 | 0.9 | 22 | 0.1 |
| No answer | 50 | 0.9 | 29 | 0.8 | 126 | 0.7 |
| **Gender** |  |  |  |  |  |  |
| Female | 4754 | 87.5 | 2882 | 83.1 | 16832 | 87.1 |
| Male | 552 | 10.2 | 505 | 14.6 | 2238 | 11.6 |
| Other | 5 | 0.1 | 4 | 0.1 | 5 | 0.0 |
| No answer | 123 | 2.3 | 77 | 2.2 | 248 | 1.3 |
| **Occupation** |  |  |  |  |  |  |
| Employed | 3707 | 68.2 | 1611 | 46.5 | 13521 | 70.0 |
| Unemployed | 171 | 3.1 | 195 | 5.6 | 374 | 1.9 |
| Student | 688 | 12.7 | 661 | 19.1 | 1266 | 6.6 |
| Seeking work | 124 | 2.3 | 176 | 5.1 | 633 | 3.3 |
| Housewife/husband | 161 | 3.0 | 214 | 6.2 | 1590 | 8.2 |
| Retired | 343 | 6.3 | 303 | 8.7 | 1051 | 5.4 |
| Other | 81 | 1.5 | 187 | 5.4 | 268 | 1.4 |
| No answer | 159 | 2.9 | 121 | 3.5 | 620 | 3.2 |
| **Education status** |  |  |  |  |  |  |
| No schooling | 3 | 0.1 | 0 | 0.0 | 32 | 0.2 |
| Primary education | 22 | 0.4 | 5 | 0.1 | 38 | 0.2 |
| Secondary education | 2002 | 36.8 | 254 | 7.3 | 2555 | 13.2 |
| Tertiary education | 3258 | 60.0 | 3129 | 90.2 | 15637 | 80.9 |
| No answer | 149 | 2.7 | 80 | 2.3 | 1061 | 5.5 |
| **Religious beliefs** |  |  |  |  |  |  |
| Religious* | 3743 | 68.9 | 1457 | 42.0 | 13011 | 67.3 |
| Not religious | 812 | 14.9 | 1521 | 43.9 | 3677 | 19.0 |
| Other | 65 | 1.2 | 57 | 1.6 | 470 | 2.4 |
| No answer | 814 | 15.0 | 433 | 12.5 | 2165 | 11.2 |
| **Relationship status** |  |  |  |  |  |  |
| Single | 1391 | 25.6 | 1411 | 40.7 | 4829 | 25.0 |
| Married | 1667 | 30.7 | 939 | 27.1 | 9138 | 47.3 |
| Cohabiting | 1338 | 24.6 | 531 | 15.3 | 2224 | 11.5 |
| Divorced/widowed | 502 | 9.2 | 303 | 8.7 | 2095 | 10.8 |
| No answer | 536 | 9.9 | 284 | 8.2 | 1037 | 5.4 |
| **No. people in household** |  |  |  |  |  |  |
| 1 | 563 | 10.4 | 467 | 13.5 | 895 | 4.6 |
| 2 | 1885 | 34.7 | 1157 | 33.4 | 4752 | 24.6 |
| 3 | 1272 | 23.4 | 694 | 20.0 | 5099 | 26.4 |
| 4 | 896 | 16.5 | 605 | 17.4 | 4067 | 21.0 |
| 5 | 190 | 3.5 | 196 | 5.7 | 1498 | 7.8 |
| More than 5 | 72 | 1.3 | 59 | 1.7 | 1375 | 7.1 |
| No answer | 556 | 10.2 | 290 | 8.4 | 1637 | 8.5 |
| **No. children in household** |  |  |  |  |  |  |
| 0 | 2708 | 49.8 | 1654 | 47.7 | 6520 | 33.7 |
| 1 | 1143 | 21.0 | 585 | 16.9 | 5812 | 30.1 |
| 2 | 696 | 12.8 | 668 | 19.3 | 3941 | 20.4 |
| 3 | 100 | 1.8 | 166 | 4.8 | 813 | 4.2 |
| 4 | 17 | 0.3 | 41 | 1.2 | 182 | 0.9 |
| 5 | 4 | 0.1 | 10 | 0.3 | 66 | 0.3 |
| More than 5 | 10 | 0.2 | 4 | 0.1 | 114 | 0.6 |
| No answer | 756 | 13.9 | 340 | 9.8 | 1875 | 9.7 |
| **Dog owner** |  |  |  |  |  |  |
| Yes | 3528 | 64.9 | 2581 | 74.4 | 10797 | 55.9 |
| No | 1836 | 33.8 | 865 | 24.9 | 8349 | 43.2 |
| No answer | 70 | 1.3 | 22 | 0.6 | 177 | 0.9 |

* Religious options included: Baha’i, Buddhism, Christianity, Candomble, Hinduism, Jainism, Jehovah’s Witnesses, Judaism, Mormonism, Islam, Paganism, Rastafarianism, Santeria, Shintoism, Sikhism, Spiritualism, Taoism, Unitarianism, and Zoroastrianism.
