# Supplementary material for: Attitudes towards free-roaming dogs and dog ownership practices in Bulgaria, Italy, and Ukraine
Source: PLoS One. 2022 Mar 2;17(3):e0252368. doi: 10.1371/journal.pone.0252368 (PMC8890656; doi:10.1371/journal.pone.0252368)
Supplement: S3 Table — (DOCX) [file pone.0252368.s006.docx]

S3 Table. Number of respondents in Bulgaria, split by regions in Bulgaria.

| **Total respondents** | **5434** | **%** |
| --- | --- | --- |
| Blagoevgrad | 119 | 2.2% |
| Burgas | 251 | 4.6% |
| Varna | 629 | 11.6% |
| Veliko Tarnovo | 181 | 3.3% |
| Vidin | 76 | 1.4% |
| Vratsa | 80 | 1.5% |
| Gabrovo | 96 | 1.8% |
| Sofia city | 1643 | 30.2% |
| Dobrich | 83 | 1.5% |
| Kardzhali | 39 | 0.7% |
| Kyustendil | 45 | 0.8% |
| Lovech | 64 | 1.2% |
| Montana | 52 | 1.0% |
| Pazardzhik | 123 | 2.3% |
| Pernik | 62 | 1.1% |
| Pleven | 149 | 2.7% |
| Plovdiv | 449 | 8.3% |
| Razgrad | 84 | 1.5% |
| Ruse | 140 | 2.6% |
| Silistra | 47 | 0.9% |
| Sliven | 76 | 1.4% |
| Smolyan | 39 | 0.7% |
| Sofia (province) | 380 | 7.0% |
| Stara Zagora | 211 | 3.9% |
| Targovishte | 55 | 1.0% |
| Haskovo | 87 | 1.6% |
| Shumen | 100 | 1.8% |
| Yambol | 50 | 0.9% |
| No answer | 24 | 0.4% |
