# Supplementary material for: Attitudes towards free-roaming dogs and dog ownership practices in Bulgaria, Italy, and Ukraine
Source: PLoS One. 2022 Mar 2;17(3):e0252368. doi: 10.1371/journal.pone.0252368 (PMC8890656; doi:10.1371/journal.pone.0252368)
Supplement: S4 Table — (DOCX) [file pone.0252368.s007.docx]

S4 Table. Number of respondents in Italy, split by regions in Italy.

| **Total respondents** | **3468** | **%** |
| --- | --- | --- |
| Abruzzo | 123 | 3.5% |
| Basilicata | 37 | 1.1% |
| Calabria | 73 | 2.1% |
| Campania | 240 | 6.9% |
| Emilia-Romagna | 281 | 8.1% |
| Friuli-Venezia Giulia | 90 | 2.6% |
| Lazio | 309 | 8.9% |
| Liguria | 137 | 4.0% |
| Lombardia | 597 | 17.2% |
| Marche | 68 | 2.0% |
| Molise | 16 | 0.5% |
| No answer | 20 | 0.6% |
| Piemonte | 281 | 8.1% |
| Puglia | 166 | 4.8% |
| Sardegna | 130 | 3.7% |
| Sicilia | 241 | 6.9% |
| Toscana | 253 | 7.3% |
| Trentino-Alto Adige | 43 | 1.2% |
| Umbria | 105 | 3.0% |
| Valle d'Aosta | 7 | 0.2% |
| Veneto | 251 | 7.2% |
| No answer | 0 | 0% |
