# Supplementary material for: Attitudes towards free-roaming dogs and dog ownership practices in Bulgaria, Italy, and Ukraine
Source: PLoS One. 2022 Mar 2;17(3):e0252368. doi: 10.1371/journal.pone.0252368 (PMC8890656; doi:10.1371/journal.pone.0252368)
Supplement: S5 Table — (DOCX) [file pone.0252368.s008.docx]

S5 Table. Number of respondents in Ukraine, split by oblasts in Ukraine.

| **Total respondents** | **19323** | **%** |
| --- | --- | --- |
| Cherkasy | 638 | 3.3% |
| Chernihiv | 419 | 2.2% |
| Chernivtsi | 415 | 2.1% |
| Dnipropetrovsk | 1702 | 8.8% |
| Donetsk | 639 | 3.3% |
| Ivano-Frankivsk | 816 | 4.2% |
| Kharkiv | 990 | 5.1% |
| Kherson | 467 | 2.4% |
| Khmelnytskyi | 587 | 3.0% |
| Kiev | 3640 | 18.8% |
| Kirovohrad | 448 | 2.3% |
| Luhansk | 199 | 1.0% |
| Lviv | 1789 | 9.3% |
| Mykolaiv | 564 | 2.9% |
| Odessa | 934 | 4.8% |
| Poltava | 744 | 3.9% |
| Rivne | 436 | 2.3% |
| Sumy | 412 | 2.1% |
| Ternopil | 513 | 2.7% |
| Transcarpathia | 362 | 1.9% |
| Vinnitsa | 632 | 3.3% |
| Volyn | 457 | 2.4% |
| Zaporizhzhia | 765 | 4.0% |
| Zhytomyr | 478 | 2.5% |
| No answer | 277 | 1.4% |
