# Supplementary material for: Attitudes towards free-roaming dogs and dog ownership practices in Bulgaria, Italy, and Ukraine
Source: PLoS One. 2022 Mar 2;17(3):e0252368. doi: 10.1371/journal.pone.0252368 (PMC8890656; doi:10.1371/journal.pone.0252368)
Supplement: S6 Table — (DOCX) [file pone.0252368.s009.docx]

S6 Table. Respondents answers to questions about ownership practices in Bulgaria, Italy and Ukraine.

|  | **Bulgaria** | **%** | **Italy** | **%** | **Ukraine** | **%** |
| --- | --- | --- | --- | --- | --- | --- |
| **Number of dog owner respondents** | **3528** |  | **2581** |  | **10797** |  |
| **No. male dogs** |  |  |  |  |  |  |
| 0 | 1168 | 33.1 | 882 | 34.2 | 3112 | 28.8 |
| 1 | 1773 | 50.3 | 1243 | 48.2 | 5206 | 48.2 |
| 2 | 347 | 9.8 | 286 | 11.1 | 1166 | 10.8 |
| 3 | 103 | 2.9 | 76 | 2.9 | 258 | 2.4 |
| 4 | 28 | 0.8 | 19 | 0.7 | 77 | 0.7 |
| 5 | 10 | 0.3 | 12 | 0.5 | 27 | 0.3 |
| More than 5 | 21 | 0.6 | 25 | 1.0 | 49 | 0.5 |
| No answer | 78 | 2.2 | 38 | 1.5 | 902 | 8.4 |
| **No. female dogs** |  |  |  |  |  |  |
| 0 | 1307 | 37.0 | 797 | 30.9 | 3468 | 32.1 |
| 1 | 1516 | 43.0 | 1215 | 47.1 | 4556 | 42.2 |
| 2 | 393 | 11.1 | 353 | 13.7 | 1179 | 10.9 |
| 3 | 102 | 2.9 | 96 | 3.7 | 413 | 3.8 |
| 4 | 38 | 1.1 | 38 | 1.5 | 131 | 1.2 |
| 5 | 28 | 0.8 | 17 | 0.7 | 62 | 0.6 |
| More than 5 | 30 | 0.9 | 28 | 1.1 | 127 | 1.2 |
| No answer | 114 | 3.2 | 37 | 1.4 | 861 | 8.0 |
| **Dog(s) registered (R) and identified (I) **** |  |  |  |  |  |  |
| Yes - all of them | 2570 | 72.8 | 2479 | 96.0 | R: 4713  I: 4482 | R: 43.7  I: 41.5 |
| No - none of them | NA*** | NA*** | 27 | 1.0 | R: 4238  I: 3647 | R: 39.3  I: 33.8 |
| Some of them | 309 | 8.8 | 46 | 1.8 | R: 641  I: 561 | R: 5.9  I: 5.2 |
| I don't know | 136 | 3.9 | 0 | 0.0 | R: 569  I: 1206 | R: 5.3  I: 11.2 |
| No answer | 513 | 14.5 | 29 | 1.1 | R: 636  I: 901 | R: 5.9  I: 8.3 |
| **Main reason for owning a dog(s)** |  |  |  |  |  |  |
| For practical reasons e.g. to guard house or for hunting | 44 | 1.2 | 21 | 0.8 | 958 | 8.9 |
| For pleasure and company e.g. as a pet or companion | 3017 | 85.5 | 2263 | 87.7 | 7631 | 70.7 |
| Practical and pleasure | 65 | 1.8 | 96 | 3.7 | 984 | 9.1 |
| Practical and other | 0 | 0.0 | 0 | 0.0 | 14 | 0.1 |
| Pleasure and other | 47 | 1.3 | 64 | 2.5 | 64 | 0.6 |
| Practical, pleasure and other | 0 | 0.0 | 4 | 0.2 | 11 | 0.1 |
| Other | 245 | 6.9 | 122 | 4.7 | 867 | 8.0 |
| No answer | 110 | 3.1 | 11 | 0.4 | 268 | 2.5 |
| **Respondents got dog(s) from: *** |  |  |  |  |  |  |
| A dog shelter | 341 | 9.7 | 986 | 38.1 | 1070 | 9.9 |
| Internet | 520 | 14.7 | 228 | 8.8 | 1311 | 12.1 |
| A shop | 214 | 6.1 | 60 | 2.3 | 232 | 2.1 |
| A breeder | 492 | 14.0 | 481 | 18.6 | 2866 | 26.5 |
| Bred my own | 208 | 5.9 | 279 | 10.8 | 716 | 6.6 |
| Found on the street | 1,252 | 35.5 | 641 | 24.8 | 3734 | 34.6 |
| From a friend/family | 1,149 | 32.6 | 590 | 22.8 | 3015 | 27.9 |
| Other | 71 | 1.6 | 113 | 4.4 | 271 | 2.5 |
| No answer | 56 | 2.0 | 21 | 0.8 | 99 | 0.9 |
| **Paid for dog(s)** |  |  |  |  |  |  |
| Yes - all of them | 1195 | 33.9 | 380 | 14.7 | 3834 | 35.5 |
| No - none of them | 1748 | 49.5 | 1821 | 70.6 | 5216 | 48.3 |
| Some of them | 473 | 13.4 | 355 | 13.8 | 1429 | 13.2 |
| No answer | 112 | 3.2 | 25 | 1.0 | 318 | 2.9 |
| **Age of dog(s) when received by respondent** |  |  |  |  |  |  |
| All puppy (<1 year) | 2683 | 76.0 | 1521 | 58.9 | 8233 | 76.3 |
| All adult (1> year) | 289 | 8.2 | 419 | 16.2 | 1140 | 10.6 |
| Some puppy, some adult | 524 | 14.9 | 639 | 24.8 | 1375 | 12.7 |
| No answer | 32 | 0.9 | 2 | 0.1 | 49 | 0.5 |
| **Owned dog(s) reproduced** |  |  |  |  |  |  |
| Yes | 780 | 22.1 | 333 | 12.9 | 2534 | 23.5 |
| No | 2674 | 75.8 | 2186 | 84.7 | 7885 | 73.0 |
| Don't know | 63 | 1.8 | 54 | 2.1 | 199 | 1.8 |
| No answer | 11 | 0.3 | 8 | 0.3 | 179 | 1.7 |
| **Total number of times owned dog(s) have reproduced** |  |  |  |  |  |  |
| Once | 521 | 14.8 | 205 | 61.6 | 1263 | 49.8 |
| Twice | 170 | 4.8 | 77 | 23.1 | 560 | 22.1 |
| Three times or more | 93 | 2.6 | 45 | 13.5 | 542 | 21.4 |
| No answer | 2744 | 77.8 | 6 | 1.8 | 170 | 6.7 |
| **Outcome of owned dog(s) puppies *** |  |  |  |  |  |  |
| Kept the puppies | 241 | 30.9 | 143 | 42.9 | 703 | 7.3 |
| Gave them to a shelter | 10 | 1.3 | 0 | 0.0 | 46 | 0.5 |
| Phoned authorities | 5 | 0.6 | 3 | 0.9 | NA | NA |
| Gave to a friend | 577 | 74.0 | 160 | 48.0 | 1858 | 19.3 |
| Sold the puppies | 162 | 20.8 | 96 | 28.8 | 1028 | 10.7 |
| Let them free in the street | 3 | 0.4 | 0 | 0.0 | 15 | 0.2 |
| Euthanised them at a clinic | 1 | 0.1 | 4 | 1.2 | 60 | 0.6 |
| Other | 133 | 17.1 | 40 | 12.0 | 1329 | 13.8 |
| No answer | 1381 | 59.6 | 4 | 1.2 | 5365 | 55.8 |
| **Prevent dog(s) from breeding** |  |  |  |  |  |  |
| Yes - all of them | 2805 | 79.5 | 2105 | 81.6 | 6024 | 55.8 |
| No - none of them | 218 | 6.2 | 217 | 8.4 | 1770 | 16.4 |
| Some of them | 124 | 3.5 | 152 | 5.9 | 1038 | 9.6 |
| Other | 45 | 1.3 | 49 | 1.9 | 241 | 2.2 |
| No answer | 336 | 9.5 | 58 | 2.2 | 1724 | 16.0 |
| **If yes, how prevent dog(s) from breeding: *** |  |  |  |  |  |  |
| Surgical neutering | 1424 | 50.8 | 1689 | 65.3 | 3828 | 35.3 |
| Restricting male and female contact | 1550 | 55.3 | 887 | 34.3 | 4052 | 37.4 |
| Other | 20 | 0.70 | 215 | 8.30 | 429 | 4.0 |
| **If no, main reason for not preventing dog(s) from breeding:** |  |  |  |  |  |  |
| Cost | 9 | 4.1 | 11 | 5.1 | 77 | 4.4 |
| A dog should reproduce at least once | 82 | 37.6 | 75 | 34.6 | 242 | 13.7 |
| Believe dog is too young to be neutered | 18 | 8.3 | 23 | 10.6 | 116 | 6.6 |
| Neutering is against religious beliefs | 6 | 2.8 | 0 | 0.0 | 19 | 1.1 |
| Neutering causes weight gain | 3 | 1.4 | 1 | 0.5 | 9 | 0.5 |
| Neutering modifies the dog’s behaviour | 10 | 4.6 | 14 | 6.5 | 60 | 3.4 |
| Neutering is a risk to the dog’s health | 32 | 14.7 | 24 | 11.1 | 286 | 16.2 |
| Other | 24 | 11.0 | 35 | 16.1 | 158 | 8.9 |
| No answer | 34 | 15.6 | 34 | 15.7 | 803 | 45.4 |
| **Respondent feeds dog(s) every day** |  |  |  |  |  |  |
| Yes | 3524 | 99.9 | 2576 | 99.8 | 10767 | 99.7 |
| No | 2 | 0.1 | 3 | 0.1 | 19 | 0.2 |
| No answer | 2 | 0.1 | 2 | 0.1 | 11 | 0.1 |
| **Respondent give dog(s) water every day** |  |  |  |  |  |  |
| Yes | 3516 | 99.7 | 2575 | 99.8 | 10724 | 99.3 |
| No | 3 | 0.1 | 4 | 0.2 | 54 | 0.5 |
| No answer | 9 | 0.3 | 2 | 0.1 | 19 | 0.2 |
| **Respondent provides provide shelter for dog(s) every day** |  |  |  |  |  |  |
| Yes | 3524 | 99.9 | 2575 | 99.8 | 10573 | 97.9 |
| No | 3 | 0.1 | 2 | 0.1 | 38 | 0.4 |
| No answer | 1 | 0.0 | 4 | 0.2 | 186 | 1.7 |
| **Respondent vaccinates dog(s)** |  |  |  |  |  |  |
| Yes | 3295 | 93.4 | 2483 | 96.2 | 8845 | 81.9 |
| No | 172 | 4.9 | 75 | 2.9 | 1668 | 15.4 |
| No answer | 61 | 1.7 | 23 | 0.9 | 284 | 2.6 |
| **Respondent allow dog(s) to go outside, in the street, unsupervised (free-roaming)** |  |  |  |  |  |  |
| Always | 366 | 10.4 | 29 | 1.1 | 382 | 3.5 |
| Sometimes | 1039 | 29.5 | 162 | 6.3 | 1764 | 16.3 |
| Never | 2082 | 59.0 | 2377 | 92.1 | 8571 | 79.4 |
| No answer | 41 | 1.2 | 13 | 0.5 | 80 | 0.7 |
| **Respondent has given up a dog(s)** |  |  |  |  |  |  |
| Yes | 33 | 0.9 | 181 | 7.0 | 676 | 6.3 |
| No | 3474 | 98.5 | 2386 | 92.4 | 9950 | 92.2 |
| No answer | 21 | 0.6 | 14 | 0.5 | 171 | 1.6 |
| **If yes, respondent:** |  |  |  |  |  |  |
| Give the dog to a shelter | 2 | 6.1 | 19 | 10.5 | 13 | 1.9 |
| Phone authorities | 0 | 0.0 | 8 | 4.4 | NA | NA |
| Give to friend | 13 | 39.4 | 133 | 73.5 | 506 | 74.9 |
| Sell | 0 | 0.0 | 9 | 5.0 | 23 | 3.4 |
| Let free | 16 | 48.5 | 0 | 0.0 | 47 | 7.0 |
| Euthanise at a clinic | 1 | 3.0 | 0 | 0.0 | 8 | 1.2 |
| Other | 0 | 0.0 | 11 | 6.1 | 65 | 9.6 |
| No answer | 1 | 3.0 | 1 | 0.6 | 14 | 2.1 |
| **If yes, reason for giving up a dog(s):** |  |  |  |  |  |  |
| Lost interest | 1 | 3.0 | 1 | 0.6 | 7 | 1.0 |
| Animal behavioural problem | 9 | 27.3 | 66 | 36.5 | 161 | 23.8 |
| Cost | 0 | 0.0 | 5 | 2.8 | 63 | 9.3 |
| Other | 13 | 39.4 | 104 | 57.5 | 306 | 45.3 |
| No answer | 10 | 30.3 | 3 | 1.7 | 139 | 20.6 |

* *Multi answer question: Percentage of respondents who selected each answer option (i.e. 100% would indicate that all respondents chose this option)*

*** Ukraine registered and identified two separate options.*

**** Answer option not available in the Bulgarian questionnaire. See S6 table.*
