# Supplementary material for: Attitudes towards free-roaming dogs and dog ownership practices in Bulgaria, Italy, and Ukraine
Source: PLoS One. 2022 Mar 2;17(3):e0252368. doi: 10.1371/journal.pone.0252368 (PMC8890656; doi:10.1371/journal.pone.0252368)
Supplement: S7 Table — (DOCX) [file pone.0252368.s010.docx]

S7 Table. Respondents answers to questions about attitudes to free-roaming dogs in Bulgaria, Italy and Ukraine.

|  | **Bulgaria** | **%** | **Italy** | **%** | **Ukraine** | **%** |
| --- | --- | --- | --- | --- | --- | --- |
| **Number of respondents** | **5434** |  | **3468** |  | **19323** |  |
| **Seen dogs free on the street** |  |  |  |  |  |  |
| Today | 3983 | 73.3 | 534 | 15.4 | 14934 | 77.3 |
| In the past week | 1144 | 21.1 | 630 | 18.2 | 3564 | 18.4 |
| In the past month | 178 | 3.3 | 557 | 16.1 | 583 | 3.0 |
| In the past year | 91 | 1.7 | 937 | 27.0 | 102 | 0.5 |
| Never | 2 | 0.0 | 650 | 18.7 | 10 | 0.1 |
| No answer | 36 | 0.7 | 160 | 4.6 | 130 | 0.7 |
| **Ever felt physically threatened by dogs in the street** |  |  |  |  |  |  |
| Yes | 1679 | 30.9 | 373 | 10.8 | 7905 | 40.9 |
| No | 3685 | 67.8 | 3060 | 88.2 | 11138 | 57.6 |
| No answer | 70 | 1.3 | 35 | 1.0 | 280 | 1.4 |
| **Ever been attacked by dogs in the street** |  |  |  |  |  |  |
| Yes | 1174 | 21.6 | 147 | 4.2 | 5129 | 26.5 |
| No | 4188 | 77.1 | 3306 | 95.3 | 13978 | 72.3 |
| No answer | 72 | 1.3 | 15 | 0.4 | 216 | 1.1 |
| **Respondent or family members been bitten by dogs in the street in the last 12 months** |  |  |  |  |  |  |
| Yes | 500 | 9.2 | 55 | 1.6 | 2900 | 15.0 |
| No | 4857 | 89.4 | 3403 | 98.1 | 16146 | 83.6 |
| No answer | 77 | 1.4 | 10 | 0.3 | 277 | 1.4 |
| **Provide care for free-roaming dogs by: *** |  |  |  |  |  |  |
| Feeding | 4911 | 90.6 | 1831 | 53.7 | 13045 | 67.5 |
| Providing water | 3847 | 71.0 | 1508 | 44.2 | 5721 | 29.6 |
| Providing shelter | 1886 | 34.8 | 647 | 19.0 | 1882 | 9.7 |
| None | 341 | 6.3 | 970 | 28.5 | 4050 | 21.0 |
| No answer | 85 | 1.6 | 484 | 14.2 | 1884 | 9.8 |
| **Respondents’ level of agreement.** |  |  |  |  |  |  |
| **I do not like free-roaming dogs being present in the streets around my home or work.** |  |  |  |  |  |  |
| Strongly agree | 1039 | 19.1 | 310 | 8.9 | 4052 | 21.0 |
| Agree | 715 | 13.2 | 364 | 10.5 | 2781 | 14.4 |
| Neither agree nor disagree | 1158 | 21.3 | 713 | 20.6 | 4669 | 24.2 |
| Disagree | 763 | 14.0 | 538 | 15.5 | 2959 | 15.3 |
| Strongly disagree | 938 | 17.3 | 1242 | 35.8 | 3114 | 16.1 |
| No answer | 821 | 15.1 | 301 | 8.7 | 1748 | 9.0 |
| **It is a good thing for the public to provide shelter for free-roaming dogs.** |  |  |  |  |  |  |
| Strongly agree | 3855 | 70.9 | 2558 | 73.8 | 15145 | 78.4 |
| Agree | 1034 | 19.0 | 584 | 16.8 | 2800 | 14.5 |
| Neither agree nor disagree | 256 | 4.7 | 175 | 5.0 | 630 | 3.3 |
| Disagree | 71 | 1.3 | 61 | 1.8 | 216 | 1.1 |
| Strongly disagree | 71 | 1.3 | 65 | 1.9 | 272 | 1.4 |
| No answer | 147 | 2.7 | 25 | 0.7 | 260 | 1.3 |
| **It is unacceptable for the public to feed free-roaming dogs.** |  |  |  |  |  |  |
| Strongly agree | 242 | 4.5 | 110 | 3.2 | 887 | 4.6 |
| Agree | 120 | 2.2 | 69 | 2.0 | 670 | 3.5 |
| Neither agree nor disagree | 272 | 5.0 | 174 | 5.0 | 1183 | 6.1 |
| Disagree | 1026 | 18.9 | 468 | 13.5 | 4536 | 23.5 |
| Strongly disagree | 3062 | 56.3 | 2383 | 68.7 | 10664 | 55.2 |
| No answer | 712 | 13.1 | 264 | 7.6 | 1383 | 7.2 |
| **It is unacceptable for the public to provide water for free-roaming dogs.** |  |  |  |  |  |  |
| Strongly agree | 182 | 3.3 | 68 | 2.0 | 621 | 3.2 |
| Agree | 89 | 1.6 | 32 | 0.9 | 471 | 2.4 |
| Neither agree nor disagree | 159 | 2.9 | 112 | 3.2 | 1113 | 5.8 |
| Disagree | 789 | 14.5 | 351 | 10.1 | 4079 | 21.1 |
| Strongly disagree | 3410 | 62.8 | 2550 | 73.5 | 11083 | 57.4 |
| No answer | 805 | 14.8 | 355 | 10.2 | 1956 | 10.1 |
| **I feel physically threatened by free-roaming dogs** |  |  |  |  |  |  |
| Strongly agree | 355 | 6.5 | 63 | 1.8 | 1957 | 10.1 |
| Agree | 328 | 6.0 | 125 | 3.6 | 2172 | 11.2 |
| Neither agree nor disagree | 612 | 11.3 | 369 | 10.6 | 2885 | 14.9 |
| Disagree | 1130 | 20.8 | 671 | 19.3 | 4890 | 25.3 |
| Strongly disagree | 2295 | 42.2 | 1956 | 56.4 | 6097 | 31.6 |
| No answer | 714 | 13.1 | 284 | 8.2 | 1322 | 6.8 |
| **Free-roaming dogs are a threat to the safety of children.** |  |  |  |  |  |  |
| Strongly agree | 513 | 9.4 | 125 | 3.6 | 2867 | 14.8 |
| Agree | 653 | 12.0 | 389 | 11.2 | 4161 | 21.5 |
| Neither agree nor disagree | 1003 | 18.5 | 746 | 21.5 | 2871 | 14.9 |
| Disagree | 1101 | 20.3 | 730 | 21.0 | 4301 | 22.3 |
| Strongly disagree | 1478 | 27.2 | 1243 | 35.8 | 3762 | 19.5 |
| No answer | 686 | 12.6 | 235 | 6.8 | 1361 | 7.0 |
| **Free-roaming dogs spread diseases.** |  |  |  |  |  |  |
| Strongly agree | 536 | 9.9 | 102 | 2.9 | 1941 | 10.0 |
| Agree | 974 | 17.9 | 373 | 10.8 | 3447 | 17.8 |
| Neither agree nor disagree | 1100 | 20.2 | 762 | 22.0 | 3470 | 18.0 |
| Disagree | 947 | 17.4 | 675 | 19.5 | 4818 | 24.9 |
| Strongly disagree | 1151 | 21.2 | 1282 | 37.0 | 4037 | 20.9 |
| No answer | 726 | 13.4 | 274 | 7.9 | 1610 | 8.3 |
| **Free-roaming dogs spread rubbish and faeces.** |  |  |  |  |  |  |
| Strongly agree | 608 | 11.2 | 157 | 4.5 | 1106 | 5.7 |
| Agree | 1114 | 20.5 | 645 | 18.6 | 946 | 4.9 |
| Neither agree nor disagree | 866 | 15.9 | 743 | 21.4 | 1902 | 9.8 |
| Disagree | 946 | 17.4 | 624 | 18.0 | 5436 | 28.1 |
| Strongly disagree | 1196 | 22.0 | 1038 | 29.9 | 8384 | 43.4 |
| No answer | 704 | 13.0 | 261 | 7.5 | 1549 | 8.0 |
| **Who should be responsible for managing free-roaming dogs (such as by providing care and/or preventing an increase in free-roaming dogs: **** |  |  |  |  |  |  |
| National government | 2425 | 44.7 | 1775 | 51.2 | 12475 | 64.6 |
| Municipality government | 4776 | 88.1 | 3201 | 92.4 | 14998 | 77.6 |
| Public veterinarians | 2639 | 48.7 | 2057 | 59.4 | 9052 | 46.8 |
| Private veterinarians | 412 | 7.6 | 249 | 7.2 | 1588 | 8.2 |
| Police | 596 | 11.0 | 240 | 6.9 | 1387 | 7.2 |
| Volunteer organisations | 2825 | 52.1 | 1517 | 43.8 | 9380 | 48.5 |
| Garbage control | 25 | 0.5 | 36 | 1.0 | 219 | 1.1 |
| Nobody | 22 | 0.4 | 7 | 0.2 | 31 | 0.2 |
| Other | 277 | 5.1 | 37 | 1.1 | 537 | 2.8 |
| No answer | 47 | 0.9 | 11 | 0.3 | 71 | 0.4 |
| **An increase in free-roaming dogs should be prevented** |  |  |  |  |  |  |
| Yes | 5177 | 95.3 | 3319 | 95.7 | 18414 | 95.3 |
| No | 111 | 2.0 | 81 | 2.3 | 461 | 2.4 |
| No answer | 146 | 2.7 | 68 | 2.0 | 448 | 2.3 |
| **If yes, how should free-roaming dogs be prevented: *** |  |  |  |  |  |  |
| Public education campaigns for responsible dog ownership | 4268 | 78.5 | 2879 | 83.0 | 12763 | 66.1 |
| School education campaigns | 3513 | 64.6 | 2356 | 67.9 | 9915 | 51.3 |
| Sanctions for abandoning dogs | 5069 | 93.3 | 3115 | 89.8 | 18027 | 93.3 |
| No answer | 94 | 1.7 | 44 | 1.3 | 417 | 2.2 |
| Other | 243 | 4.5 | 346 | 10.0 | 1029 | 5.3 |
| **Respondent prefer to see:** |  |  |  |  |  |  |
| No free-roaming dogs | 2848 | 52.4 | 2435 | 70.2 | 8740 | 45.2 |
| Fewer free-roaming dogs | 1780 | 32.8 | 841 | 24.3 | 7846 | 40.6 |
| You do not mind free-roaming dogs | 726 | 13.4 | 63 | 1.8 | 2552 | 13.2 |
| More free-roaming dogs | 4 | 0.1 | 40 | 1.2 | 21 | 0.1 |
| No answer | 76 | 1.4 | 89 | 2.6 | 164 | 0.8 |
| **If respondent prefer to see no dogs or fewer dogs on the street, how should free-roaming dogs be reduced *** |  |  |  |  |  |  |
| Remove dogs and put in shelters | 3643 | 67.2 | 1236 | 36.4 | 13349 | 69.1 |
| Catch-neuter-return of free-roamings | 3644 | 67.2 | 2088 | 61.4 | 11085 | 57.4 |
| Controlling the birth rate of owned dogs | 3811 | 70.3 | 2320 | 68.3 | 11481 | 59.4 |
| Culling | 92 | 1.7 | 56 | 1.6 | 1216 | 6.3 |
| I do not mind dogs on the street | 721 | 13.3 | 21 | 0.6 | 2388 | 12.4 |
| Other | 170 | 3.1 | 293 | 8.6 | 446 | 2.3 |
| No answer | 15 | 0.3 | 69 | 2.0 | 97 | 0.5 |

* Multi answer question: Percentage of respondents who selected each answer option (i.e. 100% would indicate that all respondents chose this option)

** Respondents allowed to choose three options for list. Percentage illustrate the percentage of respondents who selected that option
