# Supplementary material for: Attitudes towards free-roaming dogs and dog ownership practices in Bulgaria, Italy, and Ukraine
Source: PLoS One. 2022 Mar 2;17(3):e0252368. doi: 10.1371/journal.pone.0252368 (PMC8890656; doi:10.1371/journal.pone.0252368)
Supplement: S8 Table — (DOCX) [file pone.0252368.s011.docx]

S8 Table. The posterior mean values, error estimates, the 2.5 and 97.5 percentiles of the posterior distribution (CI), Rhat values and bulk and tail effective sample sizes (ESS) for Model 1 – the effect of demographic parameters on neutering status of owned dogs.

|  | **Posterior mean** | **Posterior standard deviation** | **2.5% CI** | **97.5% CI** | **Rhat** | **Bulk ESS** | **Tail ESS** |
| --- | --- | --- | --- | --- | --- | --- | --- |
| Intercept | -0.14 | 0.02 | -0.18 | -0.10 | 1.00 | 5738 | 3381 |
| *Gender* | 0.39 | 0.06 | 0.26 | 0.51 | 1.00 | 5341 | 2871 |
| *Age* | 0.14 | 0.01 | 0.11 | 0.16 | 1.00 | 6069 | 2736 |
| *Education status* | 0.25 | 0.05 | 0.16 | 0.35 | 1.00 | 5061 | 2943 |
| *Religious beliefs* | -0.41 | 0.04 | -0.50 | -0.32 | 1.00 | 5769 | 3458 |
| *Reason for dog ownership practical* | -0.72 | 0.06 | -0.84 | -0.61 | 1.00 | 5252 | 3130 |
| *Country* 1 | -0.22 | 0.03 | -0.29 | -0.16 | 1.00 | 3136 | 2917 |
| *Country* 2 | 0.61 | 0.04 | 0.54 | 0.68 | 1.00 | 3399 | 3354 |
