# Supplementary material for: Attitudes towards free-roaming dogs and dog ownership practices in Bulgaria, Italy, and Ukraine
Source: PLoS One. 2022 Mar 2;17(3):e0252368. doi: 10.1371/journal.pone.0252368 (PMC8890656; doi:10.1371/journal.pone.0252368)
Supplement: S9 Table — (DOCX) [file pone.0252368.s012.docx]

S9 Table. The posterior mean values, error estimates, the 2.5 and 97.5 percentiles of the posterior distribution (CI), Rhat values and bulk and tail effective sample sizes (ESS) for Model 2 – the effect of demographic parameters on roaming.

|  | **Posterior mean** | **Posterior standard deviation** | **2.5% CI** | **97.5% CI** | **Rhat** | **Bulk ESS** | **Tail ESS** |
| --- | --- | --- | --- | --- | --- | --- | --- |
| Threshold 1 | 0.85 | 0.02 | 0.82 | 0.88 | 1.00 | 3720 | 3284 |
| Threshold 2 | 1.86 | 0.02 | 1.81 | 1.90 | 1.00 | 4607 | 3411 |
| *Gender* | -0.05 | 0.01 | -0.06 | -0.03 | 1.00 | 5880 | 2624 |
| *Age* | -0.09 | 0.03 | -0.14 | -0.03 | 1.00 | 4525 | 2855 |
| *Education status* | 0.16 | 0.03 | 0.10 | 0.23 | 1.00 | 4052 | 3016 |
| *Religious beliefs* | 0.32 | 0.03 | 0.25 | 0.39 | 1.00 | 4295 | 2892 |
| *Reason for dog ownership practical* | 0.58 | 0.02 | 0.54 | 0.63 | 1.00 | 2289 | 2679 |
| *Country* 1 | -0.54 | 0.03 | -0.60 | -0.48 | 1.00 | 2238 | 2655 |
| *Country* 2 | 0.85 | 0.02 | 0.82 | 0.88 | 1.00 | 3720 | 3284 |
