# Supplementary material for: Attitudes towards free-roaming dogs and dog ownership practices in Bulgaria, Italy, and Ukraine
Source: PLoS One. 2022 Mar 2;17(3):e0252368. doi: 10.1371/journal.pone.0252368 (PMC8890656; doi:10.1371/journal.pone.0252368)
Supplement: S10 Table — (DOCX) [file pone.0252368.s013.docx]

S10 Table. The posterior mean values, error estimates, the 2.5 and 97.5 percentiles of the posterior distribution (CI), Rhat values and bulk and tail effective sample sizes (ESS) for Model 3 – the effect of demographic parameters and respondent experience on “*I do not like the presence of stray dogs around my home or work*”.

|  | **Posterior mean** | **Posterior standard deviation** | **2.5% CI** | **97.5% CI** | **Rhat** | **Bulk ESS** | **Tail ESS** |
| --- | --- | --- | --- | --- | --- | --- | --- |
| Threshold 1 | -0.94 | 0.01 | -0.97 | -0.92 | 1.00 | 4049 | 3359 |
| Threshold 2 | -0.36 | 0.01 | -0.38 | -0.33 | 1.00 | 4904 | 3421 |
| Threshold 3 | 0.45 | 0.01 | 0.43 | 0.47 | 1.00 | 4891 | 3234 |
| Threshold 4 | 1.03 | 0.01 | 1.01 | 1.05 | 1.00 | 5290 | 3616 |
| *Dog ownership* | 0.00 | 0.02 | -0.03 | 0.03 | 1.00 | 5292.00 | 2625.00 |
| *Gender* | -0.08 | 0.02 | -0.13 | -0.04 | 1.00 | 5752.00 | 3063.00 |
| *Age* | 0.02 | 0.01 | 0.01 | 0.03 | 1.00 | 5894.00 | 3131.00 |
| *Education status* | 0.02 | 0.02 | -0.02 | 0.06 | 1.00 | 4727.00 | 3375.00 |
| *Children in household* | 0.03 | 0.02 | -0.01 | 0.06 | 1.00 | 4924 | 3458 |
| *Threatened by dogs on the street* | 0.50 | 0.01 | 0.48 | 0.51 | 1.00 | 5102 | 3515 |
| *Been attacked by dogs on the street* | 0.05 | 0.02 | 0.01 | 0.09 | 1.00 | 4493 | 3358 |
| *Respondent or family members have been bitten by dogs on the street in last 12 months* | 0.18 | 0.03 | 0.13 | 0.23 | 1.00 | 4518 | 3180 |
| *Country1* | 0.17 | 0.01 | 0.14 | 0.20 | 1.00 | 3388 | 2792 |
| *Country2* | -0.23 | 0.02 | -0.26 | -0.20 | 1.00 | 3417 | 3051 |
