# Supplementary material for: Attitudes towards free-roaming dogs and dog ownership practices in Bulgaria, Italy, and Ukraine
Source: PLoS One. 2022 Mar 2;17(3):e0252368. doi: 10.1371/journal.pone.0252368 (PMC8890656; doi:10.1371/journal.pone.0252368)
Supplement: S11 Table — (DOCX) [file pone.0252368.s014.docx]

S11 Table. The posterior mean values, error estimates, the 2.5 and 97.5 percentiles of the posterior distribution (CI), Rhat values and bulk and tail effective sample sizes (ESS) for Model 4 – the effect of demographic parameters and respondent experience on answering an increase in stray dogs should be prevented.

|  | **Posterior mean** | **Posterior standard deviation** | **2.5% CI** | **97.5% CI** | **Rhat** | **Bulk ESS** | **Tail ESS** |
| --- | --- | --- | --- | --- | --- | --- | --- |
| Intercept | 4.09 | 0.07 | 3.96 | 4.23 | 1.00 | 4661 | 3629 |
| *Dog ownership* | 0.02 | 0.09 | -0.17 | 0.20 | 1.00 | 5643.00 | 3333.00 |
| *Gender* | 0.76 | 0.12 | 0.52 | 0.98 | 1.00 | 5277.00 | 3050.00 |
| *Age* | 0.10 | 0.04 | 0.03 | 0.17 | 1.00 | 4387.00 | 2945.00 |
| *Education status* | 0.43 | 0.10 | 0.23 | 0.62 | 1.00 | 4573.00 | 3209.00 |
| *Children in household* | -0.14 | 0.10 | -0.33 | 0.05 | 1.00 | 5149 | 2980 |
| *Threatened by dogs on the street* | 0.42 | 0.05 | 0.32 | 0.52 | 1.00 | 3860 | 3272 |
| *Been attacked by dogs on the street* | 0.07 | 0.15 | -0.21 | 0.35 | 1.00 | 4165 | 2920 |
| *Respondent or family members have been bitten by dogs on the street in last 12 months* | 0.06 | 0.20 | -0.31 | 0.45 | 1.00 | 5075 | 3416 |
| *Country1* | 0.21 | 0.09 | 0.03 | 0.40 | 1.00 | 2408 | 2787 |
| *Country2* | -0.02 | 0.10 | -0.21 | 0.17 | 1.00 | 2435 | 3022 |
