# Supplementary material for: Attitudes towards free-roaming dogs and dog ownership practices in Bulgaria, Italy, and Ukraine
Source: PLoS One. 2022 Mar 2;17(3):e0252368. doi: 10.1371/journal.pone.0252368 (PMC8890656; doi:10.1371/journal.pone.0252368)
Supplement: S12 Table — (DOCX) [file pone.0252368.s015.docx]

S12 Table. The posterior mean values, error estimates, the 2.5 and 97.5 percentiles of the posterior distribution (CI), Rhat values and bulk and tail effective sample sizes (ESS) for Model 5 – effects of demographic parameters and respondent experience on the question: Would you prefer to see: no stray dogs, fewer stray dogs, do not mind stray dogs, or more stray dogs.

|  | **Posterior mean** | **Posterior standard deviation** | **2.5% CI** | **97.5% CI** | **Rhat** | **Bulk ESS** | **Tail ESS** |
| --- | --- | --- | --- | --- | --- | --- | --- |
| Threshold 1 | 0.23 | 0.01 | 0.21 | 0.25 | 1.00 | 5604 | 3595 |
| Threshold 2 | 1.47 | 0.01 | 1.44 | 1.50 | 1.00 | 6718 | 3479 |
| Threshold 3 | 3.18 | 0.04 | 3.10 | 3.27 | 1.00 | 5042 | 3108 |
| *Dog ownership* | -0.09 | 0.02 | -0.12 | -0.05 | 1.00 | 5546.00 | 3143.00 |
| *Gender* | -0.17 | 0.02 | -0.22 | -0.13 | 1.00 | 5233.00 | 3290.00 |
| *Age* | -0.03 | 0.01 | -0.04 | -0.02 | 1.00 | 6402.00 | 3121.00 |
| *Education status* | -0.03 | 0.02 | -0.07 | 0.01 | 1.00 | 4706.00 | 3332.00 |
| *Children in household* | -0.02 | 0.02 | -0.05 | 0.01 | 1.00 | 5157 | 2954 |
| *Threatened by dogs on the street* | -0.21 | 0.01 | -0.23 | -0.20 | 1.00 | 4288 | 3245 |
| *Been attacked by dogs on the street* | -0.05 | 0.02 | -0.09 | -0.01 | 1.00 | 4156 | 3166 |
| *Respondent or family members have been bitten by dogs on the street in last 12 months* | -0.17 | 0.03 | -0.23 | -0.12 | 1.00 | 4189 | 3238 |
| *Country1* | 0.11 | 0.02 | 0.08 | 0.14 | 1.00 | 2576 | 3108 |
| *Country2* | -0.47 | 0.02 | -0.50 | -0.43 | 1.00 | 2686 | 3063 |
