# Supplementary material for: Attitudes towards free-roaming dogs and dog ownership practices in Bulgaria, Italy, and Ukraine
Source: PLoS One. 2022 Mar 2;17(3):e0252368. doi: 10.1371/journal.pone.0252368 (PMC8890656; doi:10.1371/journal.pone.0252368)
Supplement: Attachment — Submitted filename: Plos review July 2021.docx [file pone.0252368.s016.docx]

With much interest I have read and reviewed the manuscript on the study of ‘Public attitudes towards free-roaming dogs and dog ownership practices in Bulgaria, Italy, and Ukraine’. The study discusses 1) feelings, thoughts and preferences regarding stray/free roaming dogs, which are studied as *attitudes* towards these dogs, 2) the practice of neutering owned dogs and allowing owned dogs to roam, which are studied as *dog ownership practices*. The study also determines 3) *dog acquisition and abandonment practices*. Data was collected in three countries, descriptive data is presented and the three topics are studied for associations with demographic variables, including country of origin of the respondent and for associations with for instance feelings of fear for stray/free roaming dogs.

I would recommend the study to be accepted for submission by PloSOne and feel the study has value as it covers a study area that is of much relevance to dog welfare and the animal-human bond. A strong point of the study is that it was done in three countries, allowing for comparison between these countries’ respondents. Yet, the manuscript would benefit from some improvements, for which I would suggest:

***Abstract***

- Would you have enough words left to add information on the study method? The abstract now moves from aim (line 21) directly to results (line 23).
- Line 27 indicates associations between ‘public attitudes ***and*** dog ownership practices’. However, is it not 1) associations between attitudes and gender (…) and 2) associations between dog ownership practices and gender (…) that you studied?
- Line 30 and 33: are the studied variables jointly leading to higher probabilities or separately? You now use the word ‘and’. Would ‘or’ be a better choice?
- Please consider a different final sentence for the abstract. The current choice suggests something that was not studied and working cohesively towards a shared goal may require another approach than stated here.

***Introduction***

- The final sentence of the first paragraph (line 48-50) doesn’t seem to connect this paragraph to the next. It is not the *assessment* of management programs that is key to your study, right? The next paragraph is on public attitudes and their role in dog population (management). Therefore, it may help the flow of your text to stay with your topics and to choose a connecting sentence with content on these topics. (As to avoid that the reader expects the next paragraph to provide details on *assessment* of population management). A similar disruption of text flow is in line 64-65. Here ‘Reduction in numbers (…) disease control (20-22)’, hints upon the (out of scope) topic of carrying capacity. However, you wish to discuss the role of public attitudes. Perhaps rephrase to something like: ‘Without a change in demand for dogs in the community, new dogs may be bought, adopted or not prevented from moving into a community. The latter shift in dog populations may be consequential to the community’s habitat offering food, shelter, and/or social requirements’. (And check if the references still apply.)
- In line with the suggestions on terminology below, your readers will be helped by indicating somewhere in the introduction how you define/study ‘attitude’ and ‘ownership practices’ in this study, as both concepts theoretically cover a broad range of factors.

***Consistency in terminology and choice thereof***

- Please check the manuscript for consistency in terminology and choice thereof. For example, both ‘free-roaming dogs’ and ‘stray’ are used.
- Another example is the use of the word ‘social factors’ in line 37-38 of the abstract, however, is this a correct reflection of the factors that you studied?
- In the introduction: ‘strategies’ or ‘intervention (programme)s’, or both? (line 54 and 56)
- Is the OIE quote formulated as a definition? ‘there should be’ indicates that it is a list of requirements, not a definition?
- Are ‘dog abandonment level and reasons’ dog ownership practices? Are ‘acquisition reasons and manners’ dog ownership practices?
- Line 484: new terminology of ‘culture’: do you need to introduce this new terminology here? Of can you stay closer to one of the terminologies/definitions/concepts previously used/addressed in your study?

***Study sample***

- Would it be possible to add early on a clarification on the study sample: was it one study sample that was questioned on both topics (attitudes versus ownership practices)? From S2 Table 2 I would conclude that you used one sample and for some questions only the responses of the dog-owners were used. The clarification of variables (eg Table 1) provides the answer in the levels of dog owner versus non-dog owner. However, an earlier clarification would help the reader and please do consider to include the ratio. P.s. You may even reconsider using the word ‘public’ in ‘public attitudes’ in the title. The nature of the study may have attracted only a certain part of ‘the general public’. The study approach of social media targeting may also have affected who is in the sample. Like many studies (on dogs) the female-male ratio is disbalanced in this study also. These matters were discussed nicely in lines 558 onwards, and you may want to opt to adjust the title accordingly.

***Data and Methods***

- Table 1 indicates percentages of >7.5% missing values for ‘religion’, ‘do not like stray presence’, ‘feel threatened’, would you want to comment on this in the discussion, particularly on the latter two?

***Results***

- The descriptive analyses are given in percentages with the numbers of overall respondents in line 206. This facilitates readability of the text, but the downside is less accuracy of the info, which would increase by adding (N=xx of xxx).
- In the statistical analyses section subsections are titled with ‘effect of…’ this is correct as it refers to the statistical method/model used, however it may unintentionally suggest a cause-effect relationship to less experienced readers. As PloSOne is open access, you may want to consider to change the titles of the subsections. A minor thing in these titles is that there is some inconsistency in ‘on answer to’, ‘on answering’, ‘on the question’. Shorter, more aligned formatting will help the reader. Please also look at the title on page 18 again: is ‘respondent experience’ precise enough for readers to know what the section is about (and note the dot at the end of the title).
- In Table 2 the description under Model 3 would benefit from adding the topic (of stray dog presence), as was done in the other cells of this row. Some of the lower listed probabilities in this Table have three decimal places, whereas above these all have two (eg final column for children in household ‘0.593’ and see in particular Model 4 – age and below There also seems to be an indication ‘t0’ in column Model 3, 6^th^ row from below).

***Discussion***

- The first paragraph of the Discussion section on Ownership practices seems to address elements not studied. For instance ‘feeding and vaccination’. Also, was ‘quantifying the prevalence of ownership practices’ key of what you did? In addition: would you not want to mention the attitudes here also?
- The discussion could be more strongly structured. You could opt for a structure of summing up key results and then discussing the findings, after which you discuss limitation. Alternatively, you could present a key result and discuss it. You could also look at stronger starts of each paragraph. For instance, line 402 now starts with ‘There were (…)’ and line 450 with ‘This study found evidence’: such sentence parts make your writing less concise. Consequentially, it is harder for the reader to find the flow or logic between the paragraphs.
- Regarding the content of the discussion:
- Line 404 onwards indicates ‘a lack of public awareness of local shelters’, but a few sentences onwards you indicate how availability of dogs on the streets may affect ‘acquisition’. Yet, without indicating that this could also be an explanation for lesser uptake from shelters, that is if dogs are available more so ‘close by on the street’, why go to a shelter?
- Line 411 re. ‘low number of giving up’: well done that you cite the literature and possible underreporting. An addition could be to reflect a little on how your approach to gather participants may have impacted on your results? (Could these participants have been more highly engaged with their dog or with dog ownership?) You do touch upon this in lines 431 onwards and there is a nice section on it from line 500 onwards, so perhaps by restructuring the Discussion this comment becomes superfluous.
- With regard to line 411 onwards, there are some references on neutering percentages in study samples. It may put your data in perspective? (See for instance Diesel G, Brodbelt D, Laurence C. Survey of veterinary practice policies and opinions on neutering dogs. Vet Rec Open. 2010;166(15):455-458. &	Bennett PC, Rohlf VI. Owner-companion dog interactions: Relationships between demographic variables, potentially problematic behaviours, training engagement and shared activities. Appl Anim Behav Sci. 2007;102(1-2):65-84. And this ref may also be of interest: Rohlf VI, Bennett, PC, Toukhsati S, Coleman G. Why do even committed dog owners fail to comply with some responsible ownership practices? Anthrozoös. 2010;23(2):143-155.)
- Lines 450-461: and how about your study’s finding of owners thinking ‘a dog should reproduce at least once’: could lower education levels also mean lesser knowledge of possible effects (and lack thereof) of reproducing on a dog’s health and behaviour? You may have a nice opportunity here to write about attitude, knowledge, skills and behaviour and argument the statement you make later how attitudes need to be addressed as well when aiming for effective dog population management.
- Line 463 onwards: would you not want to link with mental health (next to health)? You can discuss your findings on respondents that were bitten/had family bitten and not liking the presence of stray dogs, which may indicate mental health risks for these respondents?
- Line 476: yes, and why would you anticipate this lower likeliness? Some brief words on more highly engaged dog owners being more likely to partake in studies on dogs (as discussed well in the limitation section)? Well done that you indicate percentages from other studies for comparison: an addition could be to indicate whether the study approaches differed from yours?
- Lines 538-545: You end this interesting paragraph with age as a targeting option. Would you not want to add how the attitude aspect that associated neutering in your study could be addressed specifically?
- Line 548: Mind that questionnaires do not measure on behaviour itself.

***Further suggestions***

- Abstract (line 20): ‘behaviour and outlook’, but your aim (one sentence lower) regards attitudes of dog owners and non-dog owners & practices of dog owners. Is ‘behaviour and outlook of local communities’ the right step up to your study aim? (Also in line 94 of the Introduction).
- Abstract (line 23 & 26): This study – this questionnaire: you may want to reword it (esthetical reasons only).
- Abstract (line 35): Can your findings help to ‘inform’ or ‘shape’ management interventions?
- Introduction (line 61): ‘*these* factors’: is it clear to the reader which factors?
- Introduction (line 83): ‘encouraging’ – but note that legislation may have an effect other than or next to an encouraging effect.
- If available, indications of ‘other reasons’ (eg Results line 239) would be informative when percentages are high.
