# Supplementary material for: Attitudes towards free-roaming dogs and dog ownership practices in Bulgaria, Italy, and Ukraine
Source: PLoS One. 2022 Mar 2;17(3):e0252368. doi: 10.1371/journal.pone.0252368 (PMC8890656; doi:10.1371/journal.pone.0252368)
Supplement: Attachment — Submitted filename: R1_Review_Free Roaming Dogs.docx [file pone.0252368.s017.docx]

The authors have addressed most of my comments, concerns, and questions. However, there are still a few issues with wording that should be corrected for clarity.

**Abstract**: “Assessing dog population management interventions is important to determine their long-term impact. It is essential to also determine how the attitudes and dog ownership practices within local communities may influence the efficacy of dog population management” (lines 18-21)

The phrasing and positioning of these statements still suggest to me that population management was directly assessed in this study. The statements should either a) be removed entirely or b) be edited and moved to the end of the abstract and discussed as potential future research.

**Line 95**: The red text below needs to be deleted from any methods or sections that talk about what the study did/aimed to do, because it is misleading. You absolutely can talk about future research or how your data could be used to hypothesize on the efficacy of dog population management programs, but that should be in the discussion section.

This study assesses **how the attitudes and dog ownership practices within local communities may influence the efficacy of dog population management, by gauging** attitudes towards the presence of free-roaming dogs and **of** dog ownership practices in three European countries – Bulgaria, Italy, and Ukraine.
