# Supplementary material for: Attitudes towards free-roaming dogs and dog ownership practices in Bulgaria, Italy, and Ukraine
Source: PLoS One. 2022 Mar 2;17(3):e0252368. doi: 10.1371/journal.pone.0252368 (PMC8890656; doi:10.1371/journal.pone.0252368)
Supplement: Attachment — Submitted filename: Response_to_reviewers.docx [file pone.0252368.s018.docx]

Dear Editor,

We are very grateful to both you and the reviewers for your feedback on the manuscript. We have addressed each of the comments, as outlined below:

Journal Requirements:

Please review your reference list to ensure that it is complete and correct. If you have cited papers that have been retracted, please include the rationale for doing so in the manuscript text, or remove these references and replace them with relevant current references. Any changes to the reference list should be mentioned in the rebuttal letter that accompanies your revised manuscript. If you need to cite a retracted article, indicate the article’s retracted status in the References list and also include a citation and full reference for the retraction notice.

We have reviewed the reference list to ensure it is complete and correct. We have added some references with newly included text, outlined below.

Additional Editor Comments (if provided):

This paper presents a questionnaire study carried out in three countries: Ukraine, Italy and Bulgaria. However, the author list represents research institutions in Italy and the UK and international organisations based in Austria and Belgium. Please comment on the absence of Bulgarian and Ukrainian researchers as authors of the paper. Also, please justify the choice of the three countries for the questionnaire study.

VIER PFOTEN International is an international organization with country specific offices within Bulgaria and Ukraine. We consulted with VIER PFOTEN International employees in Bulgaria and Ukraine to understand the challenges in dog population management specific to these countries, and to ensure the questionnaire was appropriate. Whilst we acknowledge these individuals in the acknowledgement section, their contribution did not merit authorship.

We have now included justification of the choice of the countries in the introduction: “We selected these focal countries due to the networks established with collaborating organisations (VIER PFOTEN International and Istituto Zooprofilattico Sperimentale dell’Abruzzo e del Molise “Giuseppe Caporale”; IZSAM) that provided local knowledge to facilitate data collection. The focal countries are culturally and environmentally distinct, allowing comparison of the collected data between different countries within Europe.”

Statistical analysis: I’m not familiar with the methods you have used for the statistical analysis, but it seems to me that a justification should be presented for the use of different approaches (Bernoulli logistic regression versus ordinal probit models) depending on which outcome variables you analysed.

We have included further detail in the statistical analysis section (i.e. that Bernoulli logistic regression are for binary outcome variables, whereas ordinal regression are used for ordered categorical outcome variables).

Results section overall: I appreciate your detailed and comprehensive presentation of the outcome of the statistical analysis, but for the paper to be accessible to the wider readership that the study topic merits, ideally this presentation should be complemented with a more straightforward summary of the results. Please consider summarizing in words the most important associations that you found, potentially as a first paragraph of the Results section. Your paper is likely to be of interest to practitioners in dog population management (e.g. shelter directors, veterinary officials), and it is important that they are able to read and understand the main findings!

We have now included a subsection “Summary of statistical associations” which reads:

“Respondents were less likely to answer that they neutered their dog(s) and more likely to answer that they allow their dog(s) to roam if they identified as (i) male, (ii) religious, (iii) owning dogs for practical reasons, (iv) young, and (v) having no schooling or primary education. Respondents were more likely answer that an increase in free-roaming dogs should be prevented if they identified as (i) female, (ii) feeling threatened by free-roaming dogs, (iii) older, and (iv) having more education. Below we report the detailed statistical findings.”

219-313 For readability, consider only including the percentage of responses, and not the n=x of y, in the written description of the results. You are also presenting these results in figures and supplementary material, and in particular the supplementary material is the best option for the additional detail.

We initially had only the percentages in the text when we first submitted the paper, but, as we included the numbers (n=x of y) as requested by reviewer 1 in the first round of feedback from our submission to this journal, we would like to keep this addition to increase the accuracy of information.

Lines 307 Is the last sentence of the figure legend not redundant?

Respondents could choose multiple answers to some of the questions in the questionnaire, whilst in others they could only choose one. We would like to indicate which results were multi answer questions, so would like to keep this sentence for clarity.

Line 303 Have you used the term CNR earlier and defined it? If it is – as a I think – used here for the first time it should be written out rather than using the abbreviation.

Thank you for spotting this – we’ve now included the full term in the introduction when we first mention reproductive control.

The authors have addressed most of my comments, concerns, and questions. However, there are still a few issues with wording that should be corrected for clarity.

**Abstract**: “Assessing dog population management interventions is important to determine their long-term impact. It is essential to also determine how the attitudes and dog ownership practices within local communities may influence the efficacy of dog population management” (lines 18-21)

The phrasing and positioning of these statements still suggest to me that population management was directly assessed in this study. The statements should either a) be removed entirely or b) be edited and moved to the end of the abstract and discussed as potential future research.

We have now removed these sentences.

**Line 95**: The red text below needs to be deleted from any methods or sections that talk about what the study did/aimed to do, because it is misleading. You absolutely can talk about future research or how your data could be used to hypothesize on the efficacy of dog population management programs, but that should be in the discussion section.

This study assesses **how the attitudes and dog ownership practices within local communities may influence the efficacy of dog population management, by gauging** attitudes towards the presence of free-roaming dogs and **of** dog ownership practices in three European countries – Bulgaria, Italy, and Ukraine.

We have now removed this and checked throughout to ensure readers are not misled.
